# The UNAIDS 90–90–90 target: a clear choice for ending AIDS and for sustainable health and development

**DOI:** 10.7448/IAS.19.1.21133

**Published:** 2016-07-15

**Authors:** Michel Sidibé, Luiz Loures, Badara Samb

**Affiliations:** UNAIDS, Geneva, Switzerland

The roadmap for ending the AIDS epidemic is clear. Combined with a stronger focus on HIV prevention, reaching the 90–90–90 target – by 2020, 90% of all people living with HIV know their HIV status, 90% of people with diagnosed HIV receive antiretroviral therapy (ART) and 90% of all people on HIV treatment achieve viral suppression – will enable us to lay the groundwork to end the AIDS epidemic by 2030 [1]. Scaled-up ART is a pillar of effective HIV prevention, as experience in different parts of the world has demonstrated that expanding the use of ART is directly correlated with declines in new HIV infections [2,3].

When the 90–90–90 target was first formulated and recommended by the UNAIDS Scientific and Technical Advisory Committee, it was met with scepticism in some quarters. However, since the launch of the 90–90–90 target in 2014, programmatic results have confirmed that the target, while ambitious, is achievable. In Malawi, a low-income country, implementation of a test-and-treat approach among pregnant women living with HIV has proven so successful that it is now seen as a stepping-stone towards universal treatment access and achievement of the 90–90–90 target [4]. Botswana, a middle-income country, is already well on its way towards the 90–90–90 benchmarks and will likely reach or exceed them before 2020 [5], and a large programme that provides ART to nearly 1000 people in community settings in Kenya and Uganda (lower middle- and low-income countries, respectively) has generated comparable results [6]. High-income countries, many of which have lagged in treatment outcomes, are also seeing improved treatment outcomes; in the United States, the proportion of people living with HIV who know their HIV status now approaches 90% [7], and the proportion of people receiving ART who are virally suppressed in the United States has risen from 72% in 2009 to 80% in 2013 [8].

Despite these promising signs, there is cause for concern, as critical steps needed to achieve 90–90–90 have yet to be taken, and key decision-makers and stakeholders have yet to display the sense of urgency needed to seize this historic opportunity to end the epidemic. Taking advantage of this unique opportunity to end the AIDS epidemic demands that we confront and overcome these obstacles.

As of 2 December 2015, only 12 countries and a large province of another country had formally adopted the World Health Organization's recommendation to initiate ART in all people living with HIV, regardless of CD4 count [9]. Although a number of additional countries have more recently adopted the recommended test-and-treat approach to ART and others are poised to do so in the coming months, the pace at which international guidance is being taken up in countries remains far too slow.

Reaching the 90–90–90 target will require roughly doubling over the next five years the number of people receiving ART. However, health systems are weak and overstretched in many countries, underscoring the need for innovative approaches to strengthen health systems. Training and empowering community health workers to assume many ART-related tasks and to bring services closer to the people who need them are an urgent necessity. UNAIDS recommends that the share of HIV-related clinical services provided in community settings must rise from 5% currently to 30% to make the achievement of 90–90–90 feasible [1].

One key gap in current treatment scale-up efforts is the failure in too many cases to reach marginalized populations, including men who have sex with men, migrants, people who inject drugs, prisoners, sex workers and transgender people [10]. In addition to implementing focused outreach and service strategies for these populations, it is also essential to remove punitive laws, policies and practices that violate human rights, increase people's vulnerability to and risk of acquiring HIV and impede utilization of services, including travel restrictions and those that block key populations’ access to services [11].

Potential threats are also emerging regarding the future availability of optimally affordable antiretroviral (ARV) medicines. Four Indian manufacturers account for roughly 70% of the ARV market in low- and middle-income countries [12]. Although local and regional manufacture of pharmaceuticals may be a longer term solution to the healthcare challenges faced by Africa and other regions, maintaining the engagement of generic producers and research and development industry will be vital for achieving the 90–90–90 target by 2020. This is especially so, given that several promising ARV products that are well advanced in the pipeline have the potential, when manufactured as generic products, to save as much as US$ 3 billion on HIV treatment costs over 10 years [13]. However, the preservation of the generic ARV market is potentially threatened by international efforts to impose, through bilateral and multilateral trade agreements, patent rules that exceed those required under international intellectual property law [14]. As a problem with a global reach and impact, AIDS demands global approaches to end this epidemic. While governments have a legitimate interest in promoting free trade and supporting their domestic industries, they must not do so in a way that imperils the ability of low- and middle-income countries to address important health needs.

In the push to achieve 90–90–90, the availability of essential funding is also a cause for concern. Especially worrisome is the flattening of international HIV assistance [15], which jump-started HIV treatment scale-up 15 years ago. Although domestic spending has stepped in to finance HIV treatment services in many countries, governments in other countries have yet to allocate sufficient domestic resources. The engagement of international donors will remain critical, especially in low-income, high-burden countries that lack the capacity to fully self-finance universal HIV treatment. At the same time that efforts are redoubled to mobilize new resources, complementary efforts will be required to improve efficiency and optimize the use of available resources. Analyses indicate that steps to maximize the efficiency of ARV procurement and HIV service delivery could help limit the costs of treatment programmes [12].

One of the targets in the sustainable development goals (SDGs), endorsed by the United Nations (UN) Member States at the 2015 UN Sustainable Development Summit, is to end the AIDS epidemic by 2030. However, the SDGs reflect a substantial broadening of the international development agenda, with ending AIDS representing only one of 169 targets. Maintaining a focus on AIDS in such a complex and crowded development agenda will be challenging. Yet, it is critical that decision-makers recognize the stakes involved in the 90–90–90 target. Following through and ending the AIDS epidemic as a public health threat will yield profound and long-lasting benefits in the form of improved productivity, averted future treatment costs and enhanced outcomes for children [1]. By the same token, a failure to build further on the achievements of the AIDS response will erase the gains made to date, allow the epidemic to rebound and vastly increase the human and financial costs associated with AIDS in future years [1]. In short, investing to end the AIDS epidemic will not only ensure that the world achieves the AIDS-specific SDG target, but also advance progress across the broad Agenda for Sustainable Development, positioning countries and their peoples to thrive in future decades.

Our “choice,” in other words, is really no choice at all. We must act now to fully leverage the preventive and therapeutic benefits of ART to lay the groundwork to end AIDS once and for all.

## Authors' informations

MS is Executive Director of UNAIDS. LL is Deputy Executive Director of UNAIDS. BS is Chief of the UNAIDS Office of Special Initiatives.
